# Plasmonic-based electrochromic materials and devices

**DOI:** 10.1515/nanoph-2023-0832

**Published:** 2024-01-04

**Authors:** Yuwei Liu, Lin Huang, Sheng Cao, Jingwei Chen, Binsuo Zou, Haizeng Li

**Affiliations:** School of Physical Science and Technology, State Key Laboratory of Featured Metal Materials and Life-cycle Safety for Composite Structures, Guangxi University, Nanning, 530004, China; School of Materials Science and Engineering, Ocean University of China, Qingdao, Shandong, 266404 China; Optics and Thermal Radiation Research Center, Institute of Frontier & Interdisciplinary Science, Shandong University, Qingdao, Shandong, 266237 China; Shenzhen Research Institute of Shandong University, Shenzhen, Guangdong, 518057, China

**Keywords:** plasmonic electrochromism, electrochromic materials, electrochromic devices, stability, nanostructure

## Abstract

The development of electrochromic (EC) materials has paved the way for a wide range of devices, such as smart windows, color displays, optical filters, wearable camouflages, among others. However, the advancement of electrochromism faces a significant hurdle due to its poor stability and limited color options. This lack of stability is primarily attributed to the substantial alteration in the dielectric properties of EC materials during cycling. Consequently, the design of advanced plasmonic materials is a key strategy to achieve a stable EC device. In this review, we provide an overview of the current state-of-the-art designs of plasmonic-based EC materials and devices. We discuss their working principles, techniques for structure/morphology engineering, doping methods, and crystal phase design. Furthermore, we explore the integration of plasmonic materials with other EC materials to create advanced EC devices. Finally, we outline the challenges that need to be addressed and present an outlook on the development of high-performance EC devices.

## 1 Introduction

Electrochromism is a fascinating phenomenon that stems from the oxidation–reduction reaction of materials under small electrical biases [1], [2], [3], [4]. This process leads to changes in optical properties, including absorption, reflection, and transparency [5], [6], [7]. Electrochromism exhibits a bistable state, allowing devices to sustain stable coloration and bleached states under open circuit conditions, thus reducing energy consumption [8], [9], [10]. These characteristics have spurred the development of various electrochromic (EC) devices, including smart windows, color displays, visual EC energy storage devices, and wearable camouflage [11], [12], [13], [14], [15], [16].

EC materials can be broadly categorized into organic materials (comprising conductive polymers and organic small molecule compounds) and inorganic materials (including metal oxides and their derivatives) [17], [18]. Organic EC materials, especially polymers, suffer from relatively poor thermal and chemical stability [19]. Doping of EC polymers results in lower-energy carrier internal transitions and the formation of polarized or bipolar states, leading to reversible changes in the optical properties of the material [20]. However, polymers are prone to fracture during the doping and dedoping processes, forming soluble low molecular weight by-products that disrupt the polymer structure [21]. Additionally, the binding force between the polymer and the substrate is weak, resulting in relatively poor thermal and chemical stability of organic materials [22]. In contrast, inorganic EC materials demonstrate better stability. However, during the EC process, ions in the electrolyte reversibly intercalate and deintercalate within the material, which can lead to structural collapse and a decrease in cyclic stability [23], [24]

To address the challenge of poor cycling stability in EC materials, the design of advanced plasmonic EC materials emerges as an effective solution [25]. Modifying the size, morphology, and dielectric environment of plasmonic materials enables the regulation of their local surface plasmon resonance, subsequently altering their optical properties. By adjusting the applied potential in plasmonic nanomaterials, control over the nucleation, growth, size, and morphology of nanoparticles is achieved [26]. Changes in the concentration of free charge carriers lead to alterations in the energy band structure of the material, affecting the absorption and reflection of light at different wavelengths and ultimately changing the material’s color [27]. Doping of plasmonic nanomaterials can also induce changes in carrier concentration, shifting plasma absorption to the near-infrared region, effectively regulating heat in sunlight. As a result, by adjusting the concentration of free carriers, independent adjustments can be made in the visible light region (10^22^ cm^−3^) [28], [29] and the infrared light region (10^18^ cm^−3^ ∼ 10^21^ cm^−3^), ultimately achieving dual-band electrochromism. Plasmonic electrochromism differs from traditional EC mechanisms in that ions do not need to be intercalated into the lattice but are only adsorbed on the surface of the EC material [30]. This circumvents lattice distortion caused by ion deintercalation, ensuring excellent cycling stability of the material [31]. For example, Xu et al. [32] reported WO_3_ film modified by Au nanoparticle–nanorods. Au nanorods were adsorbed on the surface of WO_3_, demonstrating excellent surface plasma field-enhanced absorption in the near-infrared band. The combination of plasmonic nanomaterials with traditional EC materials opens up opportunities for high-performance EC devices, particularly in military and commercial applications [33]. It is important to note that there have been no comprehensive reports on plasma-based EC materials and devices thus far. Therefore, organizing, refining, and advancing this field are critical and pressing for achieving high-performance EC devices.

In this review, we offer a detailed overview of the latest advancements and potential application areas of plasma-based EC technology. We delve into the working principles of this technology, with a particular focus on introducing several commonly used plasmonic nanomaterials for electrochromism and summarizing recent research findings. Finally, we discuss the challenges facing this technology and look forward to future research directions to promote the field’s development and provide strong support for its practical applications. This review aims to deepen the understanding of plasma EC technology, foster its widespread adoption, and contribute to its continuous enhancement.

## 2 Principles underlying the EC behaviors of plasmonic materials

The EC behavior in plasmonic materials is characterized by unique optical properties resulting from the collective oscillation of electrons within the metal lattice [34]. When an electric field is applied to a plasmonic EC material, it modulates the motion of free charge carriers, leading to changes in the local refractive index and consequent alterations in optical absorption and reflection [35], [36], [37]. This feature is particularly intriguing in the context of electrochromism, as it enables precise control of the material’s optical properties with low applied voltage [38].

The application of an electric field to a plasmonic EC material causes free carriers to migrate toward the material’s surface, resulting in an increase in the density of intrinsic free carriers [39], [40]. This heightened free carrier density plays a vital role in regulating the light absorption and emission of the material, making it suitable for various EC applications [41]. Additionally, the light modulation caused by DC bias of EC materials not only causes the change of its optical appearance but also causes the change of its high-frequency dielectric properties [42]. It is essential for the design of EC materials to consider these electrochemical reactions to ensure material stability during cycling. The dielectric environment surrounding plasmonic materials is another critical factor influencing their EC performance, influencing the movement of free charge carriers and electrochemical reactions [43]. In this section, we will discuss changes in intrinsic free carrier density, electrochemical reactions, and the influence of the dielectric environment on electrochromism in plasmonic materials.

### 2.1 Change in intrinsic free carrier density

The EC behavior in plasmonic materials results from the collective oscillation of electrons within the metal lattice [30]. The relationship between the localized surface plasmon resonance (LSPR) frequency and free carrier concentration is accurately described by the Drude–Lorentz model, which considers various factors including background polarization, permittivity of the surrounding medium, and the damping constant representing the energy loss of free carrier excitation [44], [45]

(1) $$\omega_{\mathrm{LSPR}}=\sqrt{\frac{\omega_{p}^{2}}{\varepsilon_{\infty }+2\varepsilon_{m}}-\gamma^{2}}$$

Here, *ε*_∞_ is the constant background polarization, *ε*_*m*_ is the permittivity of the surrounding medium, and *γ* is the damping constant, which signifies the energy dissipation of free carrier excitation. *ω*_*p*_ denotes the bulk plasma frequency of free carriers within the material [46]. At high-density free carrier frequency, the interaction between free carriers on the material’s surface and the surrounding dielectric environment generates a surface plasma. This relationship can be expressed as [35].

(2) $$\omega_{p}=\sqrt{\frac{ne^{2}}{\varepsilon_{0}m_{e}^{*}}}$$

In this equation, *n* is the charge carrier density, *e* is the elementary charge, *ε*_*0*_ is the free space dielectric constant, and *m*_e_^*^ is the effective mass of the electron. The LSPR primarily depends on the charge carrier density *n* and the effective mass of the electron *m*_e_^*^, as *e* and *ε*_*0*_ are constants [45]. The free carrier dielectric function is quantitatively affected by the free carrier concentration, and the plasma frequency is proportional to the square root of the free carrier concentration [44]. Consequently, adjusting the carrier concentration allows effective control over the energy and intensity of LSPR.

Moreover, adjusting the carrier concentration of the plasmonic materials enable selective spectrum adjustment to meet specific regulatory requirements for light and heat [28], [29]. At a carrier concentration of ∼10^22^ cm^−3^, LSPR exhibits absorption in the visible region (*e.g.*, Au and Ag), while at a carrier concentration of 10^18^ cm^−3^ to ∼10^21^ cm^−3^, LSPR exhibits absorption in the near-infrared region (*e.g.*, semiconductor metal oxides) [30].

### 2.2 Dielectric environments

The interaction of free carriers at the material interface with the surrounding dielectric environment leads to the generation of surface plasmons. The oscillation frequency of surface plasmon (*ω*_*SP*_) can be expressed as follows [47].

(3) $$\omega_{SP}=\omega_{p}/{\left(1+\varepsilon_{s}\right)}^{1/2}$$

When incident light irradiates the plasmonic material interface, generating surface plasma, the plasma plasmon (SPP) adheres to energy conservation principles. The wave vector (*k*_*spp*_) and wavelength of SPPs (*λ*_*spp*_) can be expressed as [48].

(4) $$k_{\mathrm{spp}}=k_{0}{\left[\varepsilon_{m}\varepsilon_{s}/\left(\varepsilon_{m}+\varepsilon_{s}\right)\right]}^{1/2}$$

(5) $$\lambda_{\mathrm{spp}}=\lambda_{0}{\left[\varepsilon_{m}\varepsilon_{s}/\left(\varepsilon_{m}+\varepsilon_{s}\right)\right]}^{1/2}$$

LSPR results from the coupling between light and finite-sized curved plasmonic nanoparticle. Taking spherical nanoparticles as an example, according to *Mie* theory, the wavelength of LSPR can be expressed as [48], [49]

(6) $$\sigma_{\mathrm{sca}}=\frac{8\pi }{3}k_{0}^{4}a^{6}\frac{{\left({\dot{\varepsilon }}_{m}-\varepsilon_{s}\right)}^{2}+{\ddot{\varepsilon }}_{m}^{2}}{{\left({\dot{\varepsilon }}_{m}+2\varepsilon_{s}\right)}^{2}+{\ddot{\varepsilon }}_{m}^{2}}$$

(7) $$\lambda_{\mathrm{LSP}}=\lambda_{p}{\left(2\varepsilon_{s}+1\right)}^{1/2}$$

Here, *σ*_*sca*_ is the polarizability of the nanoparticle, *a* is the diameter of the nanoparticle, *k*_*0*_ represents the wave vector of light in free space, *λ*_*0*_ is the wavelength of light in free space, and *ε*_*s*_ represents the dielectric function of the surrounding medium. $\varepsilon_{m}={\dot{\varepsilon }}_{m}+i{\ddot{\varepsilon }}_{m}$ represents the dielectric function of the plasma material, while *λ*_*p*_ is the plasma wavelength of the plasmonic material.

Fine-tuning the position of the plasmon resonance energy peak by manipulating the refractive index of the surrounding medium is another essential operating principle of metallic plasma [50]. In plasmonic materials, the presence of adsorbents in the electrolyte can facilitate chemisorption and metal dissolution once the applied potential surpasses a specific threshold [51]. This interaction induces changes in the morphology of metal nanoparticles, resulting in irreversible modifications to their strength, linewidth, and resonance energy [52]. The Coulomb force arising from the polarization field of the surrounding medium induces a reduction in surface charge intensity and plasma energy [53]. Notably, Zhu et al. [54] conducted a comprehensive investigation into the effect of the ambient refractive index on LSPR responses of silver-coated gold nanorods, both theoretically and experimentally. Their calculations revealed a noteworthy redshift in the low-energy plasma peak by 260 nm as the ambient refractive index increased from 1.0 to 2.0. This theoretical finding was supported by experimental results, where the Au–Ag core–shell nanorods exhibiting longitudinal wavelength of 666.5 nm experienced a redshift when varying the refractive index of the surrounding medium from 1.3494 to 1.4198.

The reflectance of nanoparticles in the surrounding medium effectively regulates the plasma resonance energy peak, as demonstrated by Yen et al. [55], in a three-dimensional finite difference time domain simulation. Their study revealed that changing the filling density of Ag NPs can produce specular reflection peaks or tilt angles at the LSPR wavelength in the reflection spectrum of the liquid mirror. Additionally, the reflection decreases when the LSPR wavelength is extinguished, and the scattering that occurs in all directions is enhanced. Therefore, it is imperative to investigate the impact of chemical reactions between the electrolyte and metal nanoparticles on plasma electrochromism. For instance, the composition and shape of the metal nanoparticles play a pivotal role in determining the wavelength of LSPR. By adjusting the ratio and size of Cu^2+^ and Bi^3+^ in the film during electrodeposition, the optical reflectance of the nanomaterials can be finely tuned. Electrodeposition in a pure Cu electrolyte results in the discontinuous growth of spheroidal Cu nanoparticles, leading to poor surface coverage. In contrast, electrodeposition in a pure bismuth electrolyte yields dendritic structures with a slow dissolution rate and a sluggish kinetic process. In a Bi–Cu electrolyte, electrochemically generated Cu^+^ ions chemically oxidize dendrite Bi atoms, filling the voids with 3 times more Cu atoms and forming a more condensed spherical shape, thereby inhibiting dendrite growth. Through the optimization of the electrolyte, the electrodeposition of Bi–Cu electrolyte enhances absorption. Simultaneously, adjusting the size distribution on the nanoscale promotes the broadening of plasmon absorption, while a similar rough feature on the order of hundreds of nanometers promotes light capture. These two effects synergistically work together to reduce reflection, increase absorption, achieve color-neutral opacity in Bi–Cu devices, ensure fast response speed, maintain high optical contrast, and provide good stability (no degradation after 1000 cycles) [56].

### 2.3 Electrochemical reactions

The free carrier mechanism provides a reasonable explanation for the EC behavior in plasmonic nanomaterials [44], [45]. Nevertheless, it remains challenging to fully account for the unexpected resonance shifts and damping observed in such materials [57]. The adsorption of substances is typically linked to plasmon resonance and is commonly observed in semiconductor metals and metallic oxides with sufficiently high free carrier concentrations [58]. The EC process in plasmonic nanomaterials typically exhibits a capacitive response, wherein the resonant interaction of free carriers within the metal gives rise to a LSPR [59]. The behavior of the metal in electrochromism is determined by the density of free electrons on the surface of metal nanoparticles, and this density undergoes changes as a result of variations in capacitive charging and discharging [32], [60]

To gain a better understanding of the unpredictable modulation of LSPR, electrochemical mechanisms are often categorized into Faraday and non-Faraday electrochemical processes [61]. Non-Faraday processes in EC materials frequently involve the adsorption and absorption of chemical substances [62]. In these processes, the charge accumulates below the interface, as it cannot pass through the metal nanoparticle solution interface, while the counter ion accumulation in the solution compensates for the potential [63]. In the context of LSPR, the Faraday process alters the refractive index of the substance, while the non-Faraday process affects the charge density on the electrode surface [50]. According to the Drude–Lorentz model, when damping is not considered, the resonant frequency is proportional to the square root of the charge carrier density (*n* ^1/2^) [44], [45]. Consequently, capacitive charging leads to a blue shift of the LSPR peak by increasing the free electron density, while discharge results in a red shift [64].

Both Faraday and non-Faraday electrochemical processes offer a robust explanation for plasmonic electrochromism [65]. Milliron et al. [66] conducted a meticulous analysis of electrochemical charging and its impact on the optical properties of modulated plasmonic materials. Specifically, they observed a nanoscale effect in Sn-doped In_2_O_3_ nanocrystals under different electrochemical conditions. During reduction, the thickness of the high refractive index shell decreased, resulting in a slight shift of the LSPR peak toward higher energy. This led to a substantial increase in the LSPR active core volume and a significant enhancement in the absorption strength of LSPR, from 40 % to 80 %. Conversely, under oxidation conditions, an increase in the thickness of the high refractive index shell caused a slight shift of the spectrum toward lower energy. The LSPR active core volume decreased from 40 % to 32 %, leading to a reduction in the absorption intensity of LSPR.

To provide a more comprehensive understanding of the mechanism of plasmonic nanomaterials in electrochromism, Kim et al. explored the intrinsic properties of individual plasmonic metal nanocrystals subjected to an applied electric field [64]. Their investigation revealed that highly charged Au nanocubes exhibit faster dispersion of radio frequencies in the range of high negative potential and maintain structural stability. This behavior is attributed to the material-specific quantum mechanical electronic structure of plasmonic nanomaterials, leading to observable changes in resonance frequency below low voltage and significant alterations in plasma scattering above the threshold voltage. The authors associate the slow blue shift observed at low voltage with these findings.

## 3 Design of nanoparticles and nanostructured plasmonic materials

The design of nanoparticles and nanostructures in plasmonic nanomaterials can enhance the material’s surface area and, concurrently, improve its optical properties through the presence of LSPR [45], [67]. Plasmonic technology has witnessed significant advancements in recent years, particularly in the realm of EC devices research [68], [69], [70]. In this section, we will provide an overview of the progress made in several common plasmonic nanomaterials.

### 3.1 Au

Au is a commonly utilized plasmonic material that has received significant attention from researchers due to its numerous synthesis methods and stable chemical properties [34], [71]. Since the 1990s, extensive research has been conducted to investigate the influence of Au nanoparticle morphology, surface charge distribution, and the surrounding environment on LSPR. Au nanoparticles exhibit a robust LSPR response in the visible region, attributed to their low resistivity, specific carrier concentration, mobility, and interband transition characteristics [72]. The combination of these properties makes Au nanoparticles particularly advantageous for applications requiring a strong LSPR response. Notably, when the carrier concentration reaches 10^22^ cm^−3^, LSPR exhibits absorption in the visible region [30]. With Au’s carrier concentration being 5.9 × 10^22^ cm^−3^, the excitation of Au nanoparticles with local surface plasmons generates a robust surface plasmon absorption band in the visible region [72], [73]. The absorption and reflection characteristics of Au nanoparticles can be effectively tuned by modifying their size, shape, thickness, and the surrounding medium, allowing for precise control of color [43]. Furthermore, adjusting the LSPR of Au nanoparticles can significantly enhance the stability of EC devices, leading to the widespread utilization of Au nanoparticles’ LSPR in the field of electrochromism.

**Figure 1:**
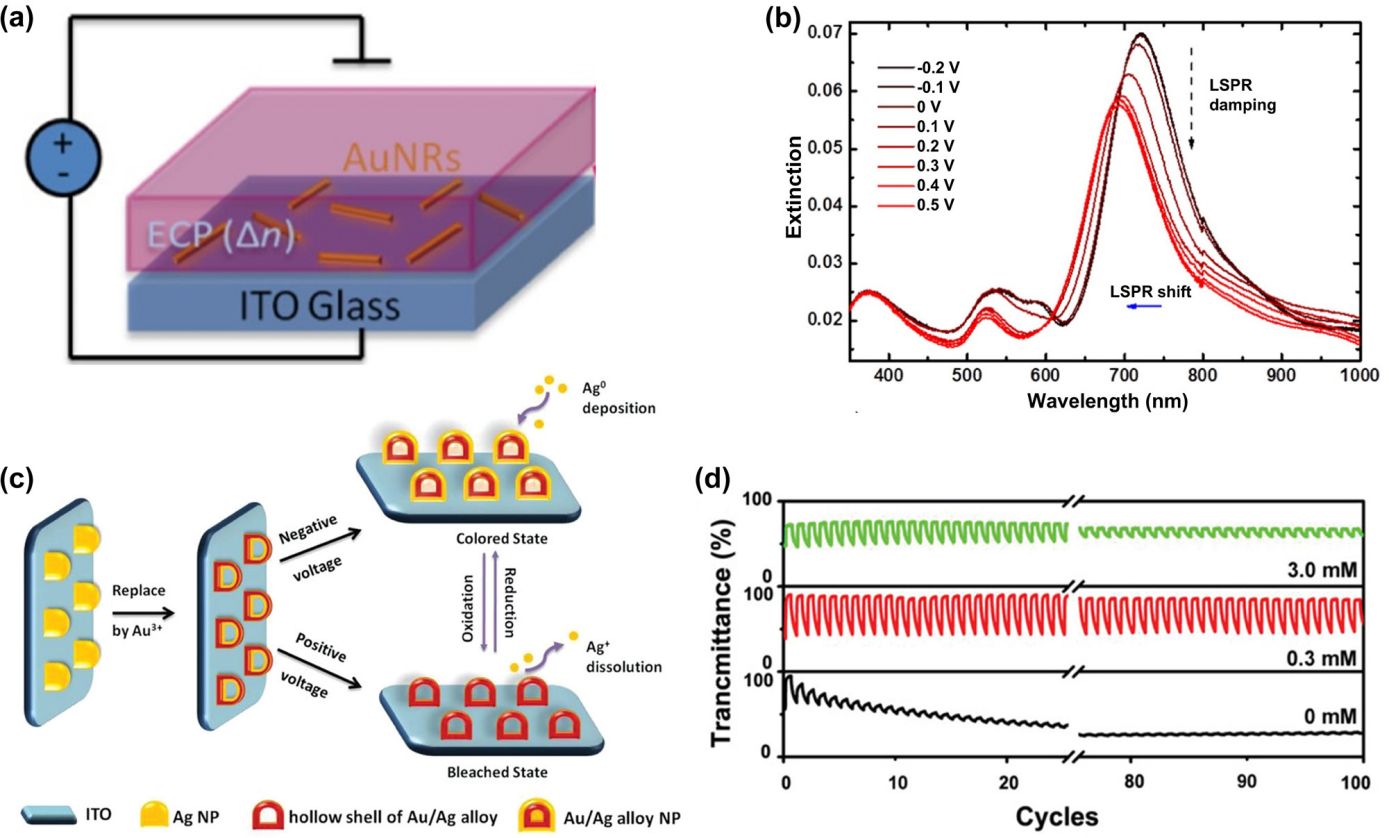
The local surface plasmon resonance of Au in electrochromic devices. (a) Experimental setup for the electro-optical plasma modulation of plasma-active polymer–metal hybrid nanomaterials. (b) The extinction spectra of AuNRs/ECP films at different voltages. The absorbance of the polymer layer was subtracted to make the contribution of LSPR to extinction more visible. Reproduced with permission [74]. Copyright 2016, American Chemical Society. (c) The process of preparing Au/Ag EC film and a schematic representation of the color-switching mechanism. (d) Transmittance response between the colored and bleached states of hollow shells of Au/Ag alloy films treated by different concentrations of HAuCl_4_ (0.3 and 3.0 mM) and the Ag nanoparticles (HAuCl_4_: 0 mM). Reproduced with permission [75]. Copyright 2019, WILEY‐VCH Verlag GmbH & Co. KGaA, Weinheim.

The LSPR wavelength of Au nanoparticles can be adjusted by modifying the dielectric environment through changes in the refractive index of the surrounding medium. For instance, Ledin et al. [74] developed an electrochemically tunable plasmon-active hybrid nanomaterial composed of a polymer–metal combination (Figure 1a). This material exhibited highly reversible optical alterations in both visible and infrared light and maintained stable LSPR modulation under the influence of external voltages. The oxidation of the polymer caused a noticeable blue shift in the LSPR peak of the Au nanoparticle rod. This shift was attributed to the increased extinction of the polaron band of alkoxy-substituted poly(3,4-propylenedioxythiophene) during the oxidation process. The polymer acted as a protective barrier, preventing the desorption and dissolution of Au nanoparticles. The amplitude of LSPR displacement decreased with an increased polymer thickness. By manipulating the refractive index of the polymer through external voltage application, a reversible shift of 27 nm in the LSPR was achieved in the Au nanorods (Figure 1b).

Additionally, the EC stability of plasmonic nanomaterials can be significantly enhanced by strategically depositing Ag seeds in specific locations through the use of Au and Ag alloys. Unlike the uncontrolled growth of undissolved Ag nanoparticles during the EC bleaching process, the metal within the EC film experiences random nucleation within the film. This random nucleation leads to uncontrollable changes in the shape of metal nanoparticles, resulting in a substantial reduction in the reversibility and stability of the EC film. To address these challenges, Li et al. [75] developed a method for creating EC films by electrically depositing Ag onto the hollow shells of Au/Ag alloys (Figure 1c). Ag atoms were preferentially deposited on the Au/Ag alloy shell through heterogeneous nucleation, resulting in a nonaggregated film that remained stable even after 100 cycles, demonstrating excellent cyclic stability (Figure 1d). Applying a negative potential led to the reduction of Ag^+^, transforming them into metallic Ag and depositing them onto the hollow shell of the Au/Ag alloy. This process continued, causing the film to exhibit transparent, blue, purple, and light red changes. The dipole mode LSPR band of the thin film gradually intensified and shifted toward shorter wavelengths. The application of a positive potential dissolved the Ag, causing the LSPR band to disappear and the film to return to a colorless state. By adjusting the grain size, both the displacement and intensity of LSPR were enhanced, resulting in a reversible shift of the plasmon band by approximately 150 nm. Consequently, the LSPR effect of Au nanoparticles not only enhances the stability of electrochromism but also enables the realization of full-color EC displays.

### 3.2 Ag

**Figure 2:**
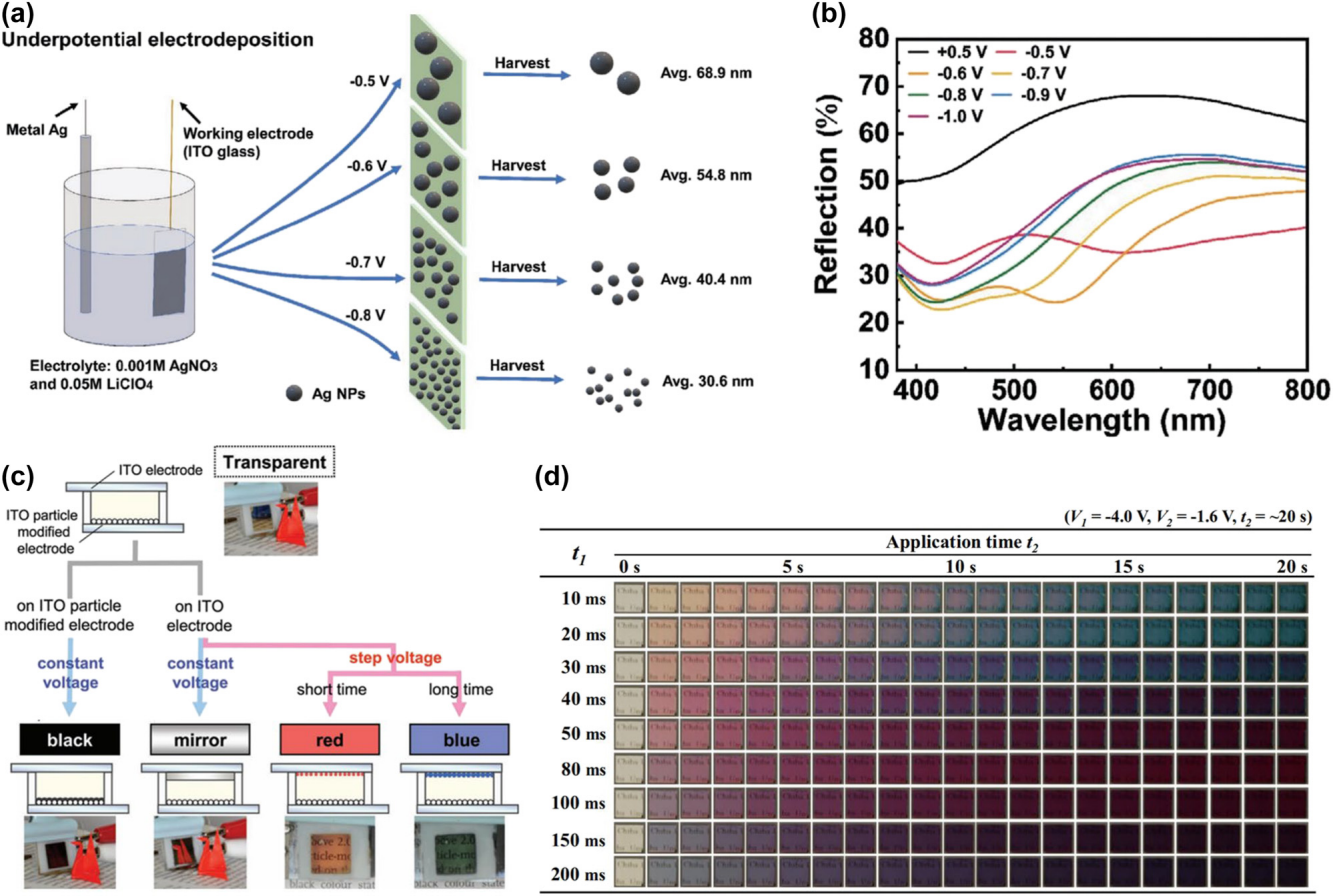
The effect of local surface plasmon resonance on electrochromic properties of Ag. (a) Schematic diagram of harvesting Ag nanoparticles of different sizes using underpotential deposition. (b) Reflectance spectra of transmission-based Ag-based EC devices at different voltages. Reproduced with permission [78]. Copyright 2022, Wiley‐VCH GmbH. (c) Scheme and photographs of two-electrode EC cell batteries in black, mirror, red, and blue states. Reproduced with permission [82]. Copyright 2013, WILEY‐VCH GmbH. (d) Photos of EC cells during Ag deposition at different nucleation times (*t*_1_). Reproduced with permission [77]. Copyright 2015, Elsevier.

Ag emerges as a preferred choice for plasmonic EC materials due to its excellent conductivity, high carrier concentration (1.07 × 10^22^ cm^−3^), and the presence of LSPR at visible frequencies [71], [72]. Ag nanoparticles demonstrate favorable features, especially in the visible and infrared plasmon regions. A noteworthy advantage lies in their lower susceptibility to quenching resulting from electron–hole pair transition coupling, particularly at higher energies. This attribute enhances the stability and applicability of Ag nanoparticles in EC applications [76]. The LSPR of Ag nanoparticles can be precisely tuned to control their optical absorption properties [77]. By manipulating the size and shape of Ag nanoparticles, it is possible to induce changes in the wavelength of the LSPR, resulting in striking variations in color [78], [79]. Therefore, Ag, as a plasmonic EC material, significantly broadens the color range for EC applications [70], [80], [81]. The reflectance spectrum can be finely adjusted by controlling the thickness of Ag nanoparticles, enabling precise management of the device’s color. For instance, Li et al. [78] manipulated Ag atoms in aqueous plasmonic EC devices by underpotential deposition to achieve size control of Ag nanoparticles and dynamic plasma color change within a 100 nm range activated by reversible voltage (Figure 2a). When the voltage is adjusted from −0.5 V to −0.8 V, the size of Ag nanoparticles shrinks (68.9 nm gradually decreases to 23.6 nm) and grows uniformly, making the LSPR peak of Ag nanoparticles significantly blue shift by 47 nm (*i.e.*, from 498 to 451 nm). The absorption of the LSPR band is relatively narrow, resulting in high color purity and a high peak extinction contrast of the film. The authors prepared a reflective Ag-ITO EC device. When the negative voltage was increased from −0.6 V to −1.0 V, the wavelength color shift of the device could reach ≈100 nm (the reflection peak gradually shifted from 780 nm to 680 nm) (Figure 2b). The LSPR wavelength of the plasma material can be effectively adjusted by altering the size and morphology of Ag nanoparticles.

The color of films can be effectively tuned by adjusting the size and shape of Ag nanoparticles in plasmonic EC materials. Norihisa and colleagues explored the various colors produced by Ag nanoparticles based on LSPR [27], [77], [82]. By applying varying nucleation pressure and growth voltage, they were able to control the size and shape of Ag nanoparticles, consequently altering the scattered light they produced (Figure 2c). At certain voltages, Ag nanoparticles formed numerous nuclei. Under different voltage conditions, nucleation and growth of Ag nanoparticles nearly ceased. Applying further voltage led to the uniform growth of Ag nanonuclei, causing shifts in the extinction bands generated by LSPR. This transformation resulted in the film transitioning from transparent to red and then to blue (Figure 2d). Therefore, by manipulating the size and shape of Ag nanoparticles, the color of the film can be precisely adjusted, making it conducive to the production of colorful EC devices. Similarly, Zhou et al. [83] employed reversible metal electrodeposition technology to directly deposit Ag nanoparticles into a traditional reversible metal electrodeposition structure that featured a layer of ordered SiO_2_ nanopore arrays as the deposition template. The tunable LSPR generated by Ag nanocolumns exhibited strong light absorption properties, allowing the color of the structure to continuously shift from brown to purple by increasing the thickness of the deposited Ag nanocolumns. Simultaneously, different color spectra could be achieved by altering the aperture and spacing of SiO_2_ nanopore templates, demonstrating the effectiveness of color design based on LSPR.

### 3.3 Cu

While Au and Ag are known to possess excellent plasmonic EC properties, their high cost poses limitations on their practical applications in both industry and commerce [69]. In contrast, Cu emerges as an economically viable and highly conductive plasmonic material with abundant availability, demonstrating robust LSPR characteristics within the visible and near-infrared spectral regions [84]. Cu nanoparticles offer notable catalytic activity and size-dependent optical absorption [85]. These characteristics make Cu nanoparticles compelling choice for specific applications in electrochromism. Therefore, investigating the utilization of Cu nanomaterials in plasmonic electrochromism holds significant importance.

**Figure 3:**
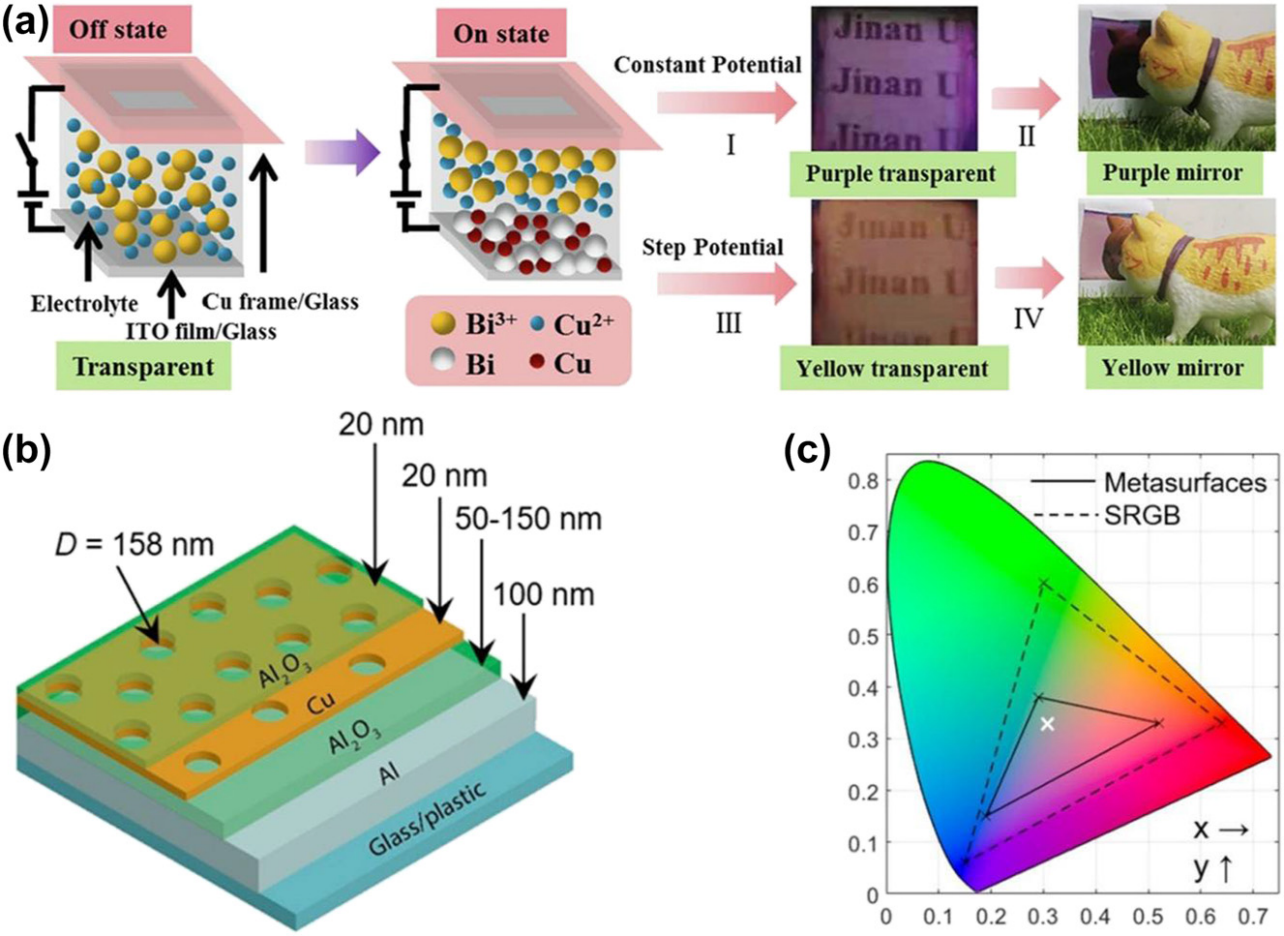
The color formed by the local surface plasmon resonance of Cu in electrochromic devices. (a) Reversible metal electrodeposition devices achieve multiple color states at different voltages. Reproduced with permission [86]. Copyright 2022, Elsevier. (b) Structural layout of metal–insulator–nanopore array metasurfaces based on Cu thin films. (c) CIE 1931 chromaticity space shows a comparison between metasurface and standard RGB. Reproduced with permission [87]. Copyright 2017, American Chemical Society.

The absorption and scattering of light can be effectively regulated by adjusting the shape and size of Cu nanocrystals. He et al. [86] controlled the ratio of Cu^2+^ and Bi^3+^ in the electrolyte and size of deposited nanoparticles on ITO through electrodeposition, allowing precise tuning of the optical reflectance of nanoionparticles (Figure 3a). This control facilitates accurate management of the resulting color change. Moreover, by adjusting the electrodeposition potential values, such as *V*_1_, *V*_2_, and *V*_3_, along with their corresponding durations, both the potentiostatic and step potential methods can be employed to manipulate the size of Bi–Cu nanoparticles. The potentiostatic approach enables an increase in the size of Bi–Cu nanoparticles from 73.72 nm to 108.57 nm, while the step potential method achieves a size range from 36.84 nm to 78.41 nm. This allows the adjustment of visible region to achieve various optical states, including transparency, purple transparency, purple mirror, yellow transparency, and yellow mirror. Importantly, devices created using this approach demonstrate exceptional cycle stability and rapid response speeds. Additionally, Melepurath Deepa et al. [85] have improved the light absorption of thin films by introducing the Cu fiber plasmonic effect. They enhanced the EC properties of poly(3,4-ethylenedioxythiophene) (PEDOT) by creating a PEDOT/Cu hybrid film, capitalizing on the light-absorbing capacity of Cu nanofibers and the synergistic effect of their high electrical conductivity. The extensive surface area of Cu fibers offers a larger number of active sites for the hybrid membrane, resulting in enhanced ion absorption. The surface plasmon peak of Cu nanoparticles coincides with the π–π absorption of PEDOT, leading to a reduction in the band gap and a blue shift in LSPR. Additionally, Cu nanoparticles serve as conductive bridges within the polymer, reducing diffusion impedance and enhancing electron and ion transport. As a result, the exceptional electrical conductivity of Cu fibers and the combined plasmonic effects augment the film’s absorption properties, leading to a substantial improvement in its EC performance.

Leveraging the plasmonic attributes of Cu allows for an expansion of the color range in EC devices. Xiong et al. [87] have proposed a high chromaticity plasmonic metasurface based on a thin film structure featuring a metal–insulator–nanopore array (Figure 3b). When the thickness of the Cu film is set at 30 nm, short-range order in the aperture couples with the surface plasmon, producing distinctive characteristic peaks in the extinction spectra. Reducing the thickness of the Cu film to 20 nm induces a redshift in the resonance. The authors employ a combination of Fabry–Perot interference (cavity mode) and surface plasmon to achieve highly resonant polarized reflection. This integration of Fabry–Perot interference and surface plasmon becomes particularly evident when the Cu film thickness is 30 nm, resulting in pronounced characteristic peaks in the extinction spectra. Reducing the thickness of the Cu film to 20 nm triggers a redshift in the resonance (Figure 3c). Consequently, the authors leverage a blend of Fabry–Perot interference and surface plasmon to attain a state of highly resonant polarized reflection.

### 3.4 Metal oxides

Metal oxides, such as niobium oxide, titanium dioxide, and tungsten oxide, have the ability to modify their optical absorption and reflection characteristics by adjusting their chemical composition, specifically by tuning the plasmon resonance frequency [88], [89], [90]. This adjustment in the composition is achieved through processes such as electrochemical deposition and ion adsorption, resulting in changes in the LSPR response of these materials [91]. This selective tuning of visible and infrared light absorption is used to enhance EC stability [26].

In 2011, the Milliron group successfully optimized specific wavelengths within the near-infrared spectrum by introducing tin-doped indium oxide (ITO) nanocrystals into niobium oxide glass and adjusting the doping levels of ITO nanocrystals within niobium oxide. Their results demonstrated that the plasmon resonance of thin films can be dynamically adjusted through entirely reversible electrochemical doping, allowing for the manipulation of semiconductor LSPR characteristics. This marked the official commencement of research into plasmonic EC technology. Subsequently, they conducted a series of experiments in which they modulated visible and infrared light by doping niobium oxide with ITO nanocrystals. Under different bias, niobium oxide primarily regulates visible light, but when ITO plasmonic nanocrystals are subjected to a voltage of 2.3 V, the free carrier concentration reaches 1.9 × 10^21^ cm^−3^, selectively regulating near-infrared light. The insertion of ITO nanocrystals formed covalent bonds with niobium oxide, leading to the reorganization of the niobium oxide structure, enhancing ion transport, and significantly improving the EC stability and optical contrast of the film [92].

**Figure 4:**
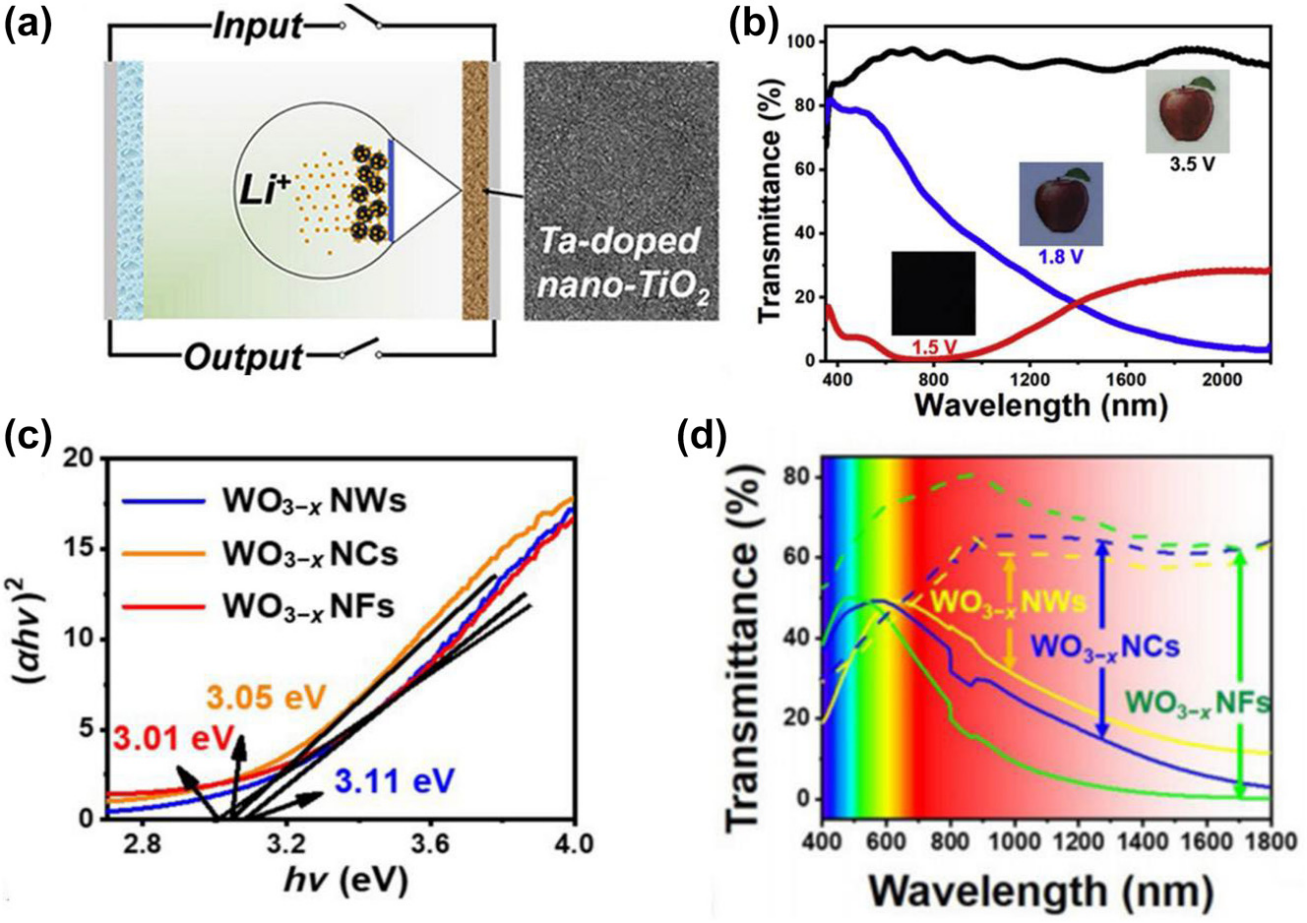
Effect of doping on properties of metal oxides. (a) Mechanism and morphology of Ta-doped TiO_2_ nanocrystals. (b) Light transmittance of Ta-doped nano TiO_2_ film and corresponding digital photos. Reproduced with permission [8]. Copyright 2018, Elsevier. (c) Bandgap calculations of three WO_3−*x*_ nanostructures. (d) Transmission spectra of WO_3−*x*_ NWs, WO_3−*x*_ NCs, and WO_3−*x*_ NFs in 1.0 M LiClO_4_/PC electrolyte at −1.5 V and 1.0 V. Reproduced with permission [65]. Copyright 2023, Tsinghua University Press.

In metal oxides, the combination of plasmonic and traditional polaron electrochromism can result in dual-band EC modulation. Cao et al. [8] synthesized colloidal tantalum-doped anatase nanocrystals using fluoride-assisted methods, allowing for independent control of visible and infrared light during EC processes (Figure 4a). At lower bias voltages, Li^+^ attaches to the surface of nanocrystals, leading to the generation of free carriers in the conduction band of TiO_2_. The resonant coupling of these free carriers creates LSPR, resulting in the absorption of near-infrared light while maintaining high transmittance of visible light. At higher bias levels, when Li^+^ ions are incorporated into TiO_2_, they almost completely block visible and infrared light (Figure 4b). Capacitive charging-induced absorption in the near-infrared enhances the stability of the device, resulting in light modulation losses of 0.2 % at 550 nm and 6.0 % at 1600 nm, bistable effects (transmittance varying by less than 1.5 % in both colored and bleached states under open circuit conditions), and a high dynamic range (89.1 % at 550 nm and 81.4 % at 1600 nm). Huang et al. [65] prepared tungsten oxide (WO_3−*x*_) nanoflowers (NFs) by controlling the precursor concentration and adjusting the lattice fringe spacing of WO_3−*x*_ nanostructures (Figure 4c). The significant oxygen vacancies in WO_3−*x*_ reduce its bandgap (*E*_*g*_), leading to an increased free carrier density. Additionally, the intricate multi-dimensional structure of WO_3−*x*_ NFs offers more LSPR active sites, resulting in multiple light absorption and enhancing the LSPR effect. WO_3−*x*_ NFs exhibit robust LSPR absorption and excellent near-infrared blocking capabilities, functioning in three modulation modes (bright, cool, and dark) (Figure 4d). Furthermore, WO_3−*x*_ NFs boast a short response time (*τ*_*b*_/*τ*_*c*_ = 1.54/6.67 s) and excellent EC cycle stability, with a capacity retention rate of 97.75 % after 4000 s. Therefore, doping plasmonic nanomaterials to regulate their free carrier concentration, combined with the design of material structure/morphology, can achieve dual-band electrochromism and improve EC stability.

## 4 Integrating plasmonic materials with other EC materials to design advanced EC devices

The integration of plasmonic materials with traditional EC materials presents a promising strategy for tuning the LSPR of plasmonic materials and enhancing the stability of EC materials [93], [94], [95]. Plasmonic materials can effectively be combined with various traditional EC materials such as WO_3_, TiO_2_, NbO, as well as polymers like PANI and PEDOT, etc., to create advanced EC devices.

### 4.1 EC oxides

Plasmonic materials can be integrated with EC oxides to develop advanced EC devices. The LSPR can be controlled by adjusting the geometrical parameters of the metal nanostructure, the periodicity of the plasma array, and the dielectric constant of the metal nanostructure itself and its surrounding medium [49], [96]. However, plasma nanostructures typically have fixed optical properties once designed and manufactured, posing a challenge.

**Figure 5:**
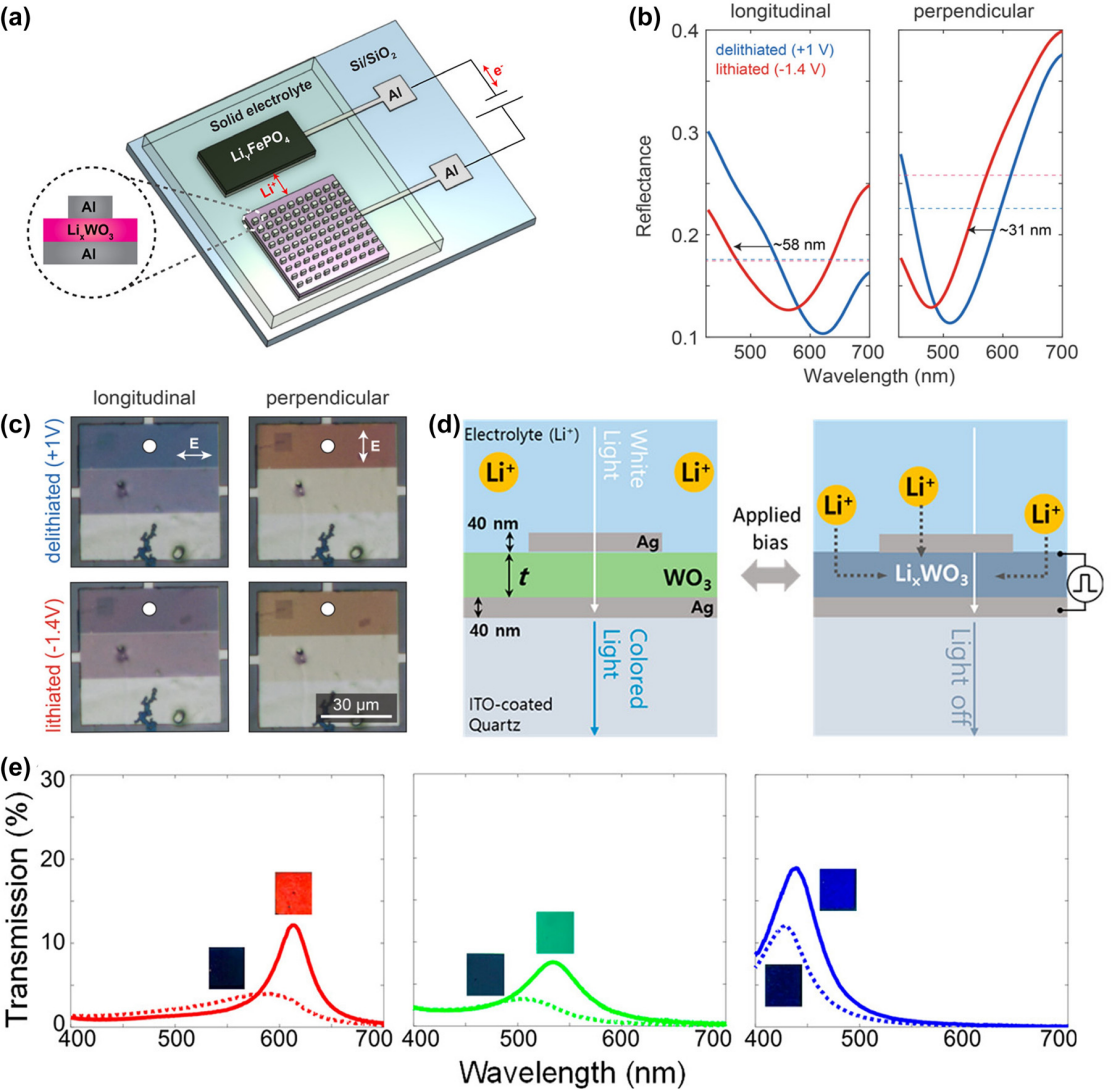
Regulation of band gap in plasma electrochromic devices. (a) Schematic diagram of a gap plasma device with tunable structural color based on EC material WO_3_. (b) Measured reflection spectra of longitudinal polarization (left) and vertical polarization (right). The dashed line shows the average reflectance over the experimental spectral range. (c) Optical microscope images of a gap plasma device based on EC material WO_3_ in delithiated (top) and lithiated (bottom) states. Reproduced with permission [68]. Copyright 2019, American Chemical Society. (d) Schematic diagram of transmission EC device. With the increase of applied potential, it is difficult for incident light to penetrate the device. t is the thickness of WO_3_ film. (e) Experimental spectra of transmitted light in red, green, and blue, with solid and dashed lines indicating ON and OFF coloring states, respectively. Reproduced with permission [98]. Copyright 2020, American Chemical Society.

This challenge can be overcome by integrating plasmonic materials with EC materials. When combined with EC oxides as the dielectric layer, a metal–dielectric–metal gap plasma resonator can be created, which can be dynamically tuned. By applying voltage to change the refractive index of the EC material, and thus altering the dielectric constant of the surrounding plasmonic material, the spectral characteristics of the plasma device can be dynamically controlled [47], [97]. In 2019, Li et al. [68] utilized WO_3_ thin films as spacer layers to construct an Al/Li_*X*_WO_3_/Al (0 < *X* < 0.2) metal/insulator/metal gap plasma structure (Figure 5a). Under electrochemical action, Li^+^ is embedded in the WO_3_ lattice, which becomes Li_*X*_WO_3_, and the refractive index *n* changes from 2.1 to 1.9. This alteration in the refractive index affects the dielectric environment of the plasmonic material of aluminum (Al), leading to a 58 nm shift in the resonant wavelength of the plasma (Figure 5b and c). However, Li et al.’s approach did not fully meet the requirements for display applications in terms of color purity. In 2020, Lee et al. [98] developed a transmission-type device through straightforward thin film deposition, with WO_3_ thin films placed between two 40 nm thick Ag layers (Figure 5d). This gap plasma resonator, operating based on Fabry–Perot type resonance, offered significant advantages in terms of color purity due to its sharp and linear spectrum. It achieved high purity on/off color transitions with a transmittance modulation of up to 4.04 (Figure 5e).

Eaves-Rathert et al. [99] employed TiO_2_ as a dielectric layer between Ag nanocolumns and an Al backplane to create a gap plasma structure. The resonance condition of the plasmon on the back propagation gap’s surface determined destructive interference with increasing wavelength. This led to the concentration of the electric field at the gap between the column and the backplane at the minimum reflectivity frequency. As a result, as the diameter of the light column increased, the reflected color shifted from gold to brown. When the film transitioned from anatase TiO_2_ to electrochemical Li_0.5_TiO_2_ (LTO), the wavelength at the minimum reflectance of the Ag nanocrystals with diameters of 90, 80, 70, and 60 nm shifted by 108, 59, 39, and 37 nm, respectively. This demonstrated that the gap plasma resonator could be constructed to integrate plasmonic material and EC oxide, with the spectrum being dynamically regulated by changing the dielectric environment of the plasmonic material.

**Figure 6:**
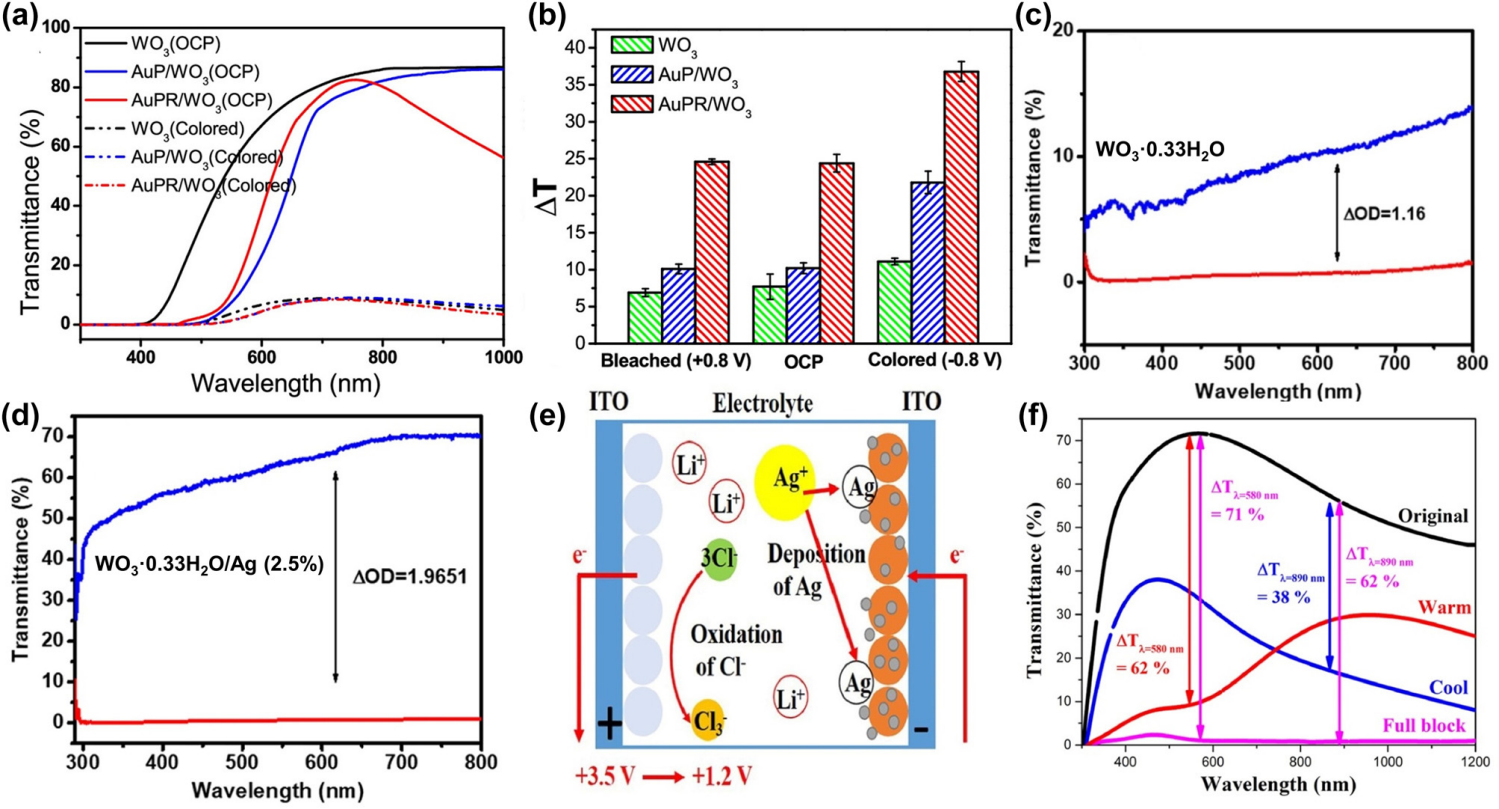
Effects of metal plasma materials on electrochromic properties in combination with metal oxides. (a) Transmission spectra of WO_3_, AuP/WO_3_, and AuPR/WO_3_ films in OCP and colored states. (b) Temperature increase on WO_3_, AuP/WO_3_, and AuPR/WO_3_ film coated FTO surfaces after 300 s irradiation with a 915 nm laser in 3 mL phosphate-buffered solution. Reproduced with permission [32]. Copyright 2018, American Chemical Society. (c) Transmission spectra of WO_3_·0.33H_2_O films in colored and bleached states. (d) Transmission spectra of WO_3_·0.33H_2_O/Ag (2.5 %) films in colored and bleached states. Reproduced with permission [100]. Copyright 2021, Acta Materialia Inc. (e) Schematic diagram of an EC device consisting of a TiO_2_ EC film, LTA electrolyte, and gallium-doped zinc oxide electrode in warm mode. (f) Transmission curves of an EC device consisting of a TiO_2_ EC film, LTA electrolyte, and gallium-doped zinc oxide electrode under four modes. Reproduced with permission [101]. Copyright 2023, Elsevier Ltd.

For EC oxides, integrating them with plasmonic materials can enhance the light absorption of EC oxides and expanding the optical modulation range. Xu et al. [32] developed an EC photothermal film, denoted as AuPR/WO_3_, by incorporating Au nanoparticles and Au nanorods into a three-dimensional honeycomb porous WO_3_ film. In this structure, the WO_3_ EC film serves as the substrate, while Au nanoparticles act as anchor points, and Au nanorods amplify the near-infrared surface plasmonic resonance. The AuPR/WO_3_ sample exhibits significant absorption in both the visible and near-infrared bands when in its colored state (−0.8 V). This is achieved through the optical modulation of WO_3_ in the visible spectrum and the LSPR of the metal nanostructures in the near-infrared spectrum. In the open circuit potential state, AuPR/WO_3_ exhibits enhanced near-infrared light blocking due to these SPR characteristics. Additionally, the transmittance of AuPR/WO_3_ can be adjusted by applying different bias voltages (Figure 6a). Importantly, AuPR/WO_3_ demonstrates enhanced photothermal conversion, with the solution temperature change exceeding the sum of temperature changes generated by AuP/WO_3_ and WO_3_ alone after 300 s of laser irradiation (Figure 6b). This enhanced photothermal conversion is attributed to the strong absorption of the near-infrared plasmon resonance at the interface between AuPR and the WO_3_ substrate, with local plasmon resonance amplifying the local field, thereby improving WO_3_ absorption and photothermal conversion. This EC photothermal film holds promise for use in the next generation of smart windows capable of converting solar energy into thermal energy for building heating.

Furthermore, integrating EC oxides with plasmonic materials enhances the light absorption of EC oxides. Deonikar et al. [100] prepared WO_3_·0.33H_2_O/Ag(*x*%) nanocomplexes with varying Ag loads through a hydrothermal method. By optimizing the loading capacity of Ag nanoparticles (the weight ratio of AgNO_3_ ranging from 0 to 20 %), a wider optical modulation range could be achieved. WO_3_·0.33H_2_O/Ag (2.5 %) films exhibited the broadest optical modulation range, reaching up to 67.11 % at 633 nm, surpassing WO_3_·0.33H_2_O without Ag (with an optical modulation range of about 10 %) and other Ag-loaded WO_3_·0.33H_2_O/Ag(*x*%) films. In Figure 6(c and d), the notable increase in transmittance change is explained by the elimination of an electron from the electrochromic layer. WO_3_·0.33H_2_O loaded with Ag nanoparticles demonstrates a significant increase in surface area due to the presence of more defects and the fine anchoring of Ag nanoparticles on the surface of WO_3_·0.33H_2_O. This results in a substantial change in transmittance during the electrochromic process. When plasmonic materials are integrated with EC oxides, their respective dimming regions can be combined to create dual-band EC devices capable of selectively regulating visible and near-infrared wavelengths. Zhang et al. [101] achieved this by utilizing the EC function of a TiO_2_ film to control near-infrared light and Ag nanoparticles to modulate visible light through LSPR. They used gallium-doped zinc oxide as an ion storage membrane and LiClO_4_ + TBACl + AgNO_3_ (LTA) as an electrolyte. In the visible light spectrum, Ag^+^ in the electrolyte was deposited on the TiO_2_ surface through a two-stage potential to form Ag nanoparticles, stimulating the LSPR effect, causing all incident light to be either reflected or absorbed (Figure 6e). In the near-infrared spectrum, Li^+^ was embedded into the TiO_2_ lattice structure, causing the TiO_2_ EC film to exhibit a colored state, effectively blocking near-infrared light. Furthermore, by increasing the surface roughness of the TiO_2_ film, the light scattering effect was maximized, resulting in near 0 % transmittance in the fully block mode (Figure 6f).

### 4.2 EC molecules and polymers

Integrating plasmonic materials with EC molecules and polymers offers the ability to tune the light absorption and reflection of EC devices. This integration involves incorporating EC molecules and polymers into the plasmonic structure, enabling the tuning of plasmon resonance by applying voltage to change the refractive index of these EC components, subsequently altering the dielectric constant of the surrounding environment.

**Figure 7:**
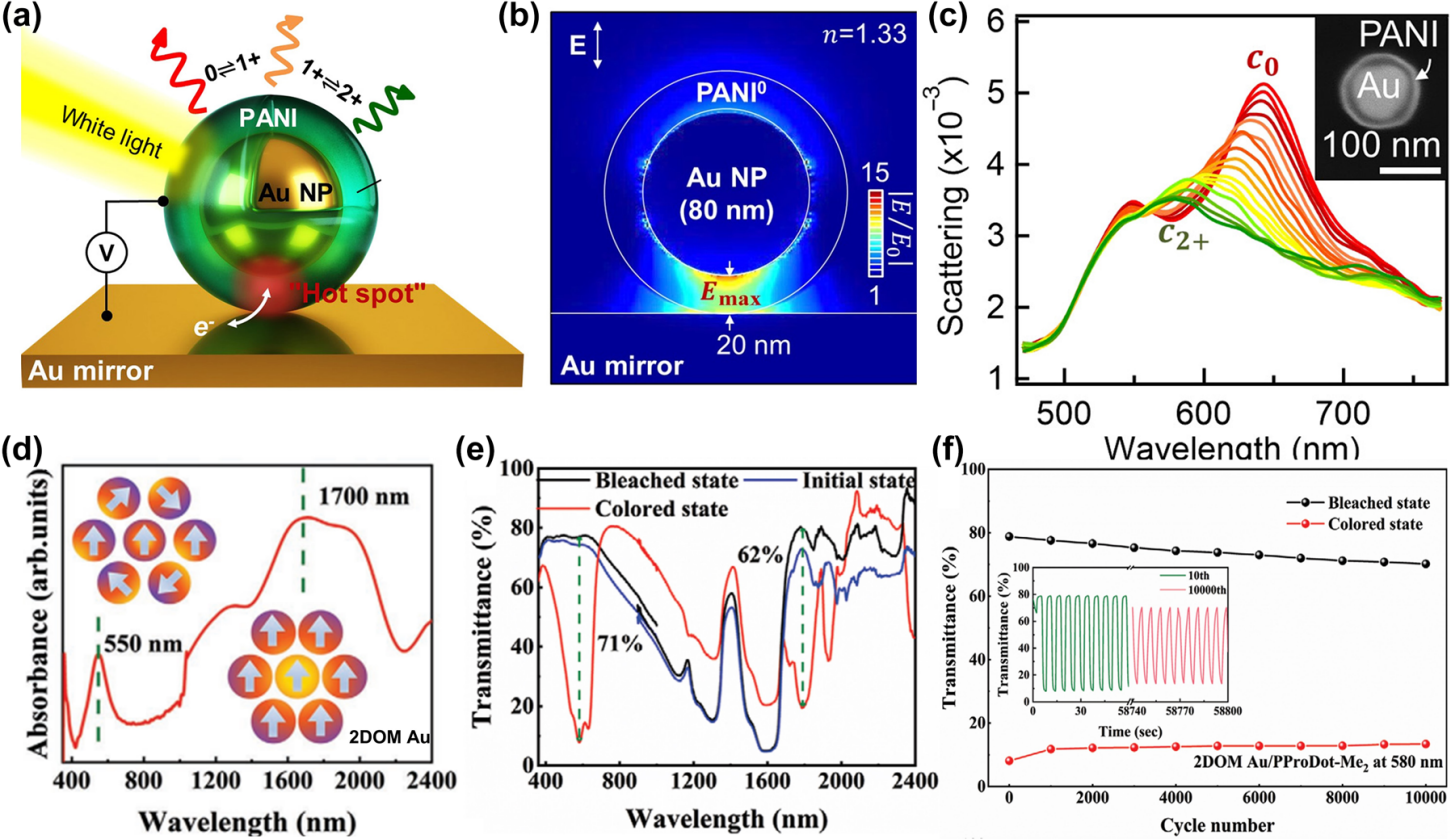
Effects of metal plasma materials on the properties of electrochromic polymers. (a) Schematic of an EC nanoparticle-on-mirror constructs (eNPoM) formed from Au nanoparticles (Au NPs) encapsulated in a conductive polymer shell. (b) In the reduced state of the polyaniline shell, the optical near-field enhancement of eNPoM shows a hot spot in the gap between the Au core and the Au mirror. (c) The scattering spectrum of a single eNPoM when the voltage is swept from −0.2 V to 0.6 V at a sweep speed of 50 mV/s. Reproduced with permission [102]. Copyright 2019, The Authors. (d) Absorption spectra and charge density maps of the 2DOM Au layer. (e) Transmission spectra of 2DOM Au/PProDot-Me_2_ films in initial, colored and colored states. (f) Transmittance evolution of 2DOM Au/PProDot-Me_2_ films at 580 nm wavelength during 10,000 CA cycles. Reproduced with permission [104]. Copyright 2023, Wiley‐VCH GmbH.

Stockhausen et al. [102] reported on the changes in the LSPR of Au nanoparticle arrays coated with PEDOT as a function of the polymer’s state. When a voltage of 0.6 V is applied, the LSPR of Au nanoparticles covered by PEDOT shifts dramatically from a spectral peak at 760 nm–685 nm (blue shift). Conversely, a voltage of −1.0 V shifts the peak from 685 nm to 877 nm (red shift). The total spectral offset between these two voltages is a substantial 192 nm, covering nearly half of the visible spectrum. Similarly, Peng et al. [103] created an EC electrode by encapsulating Au nanoparticles in a PANI shell and then depositing the Au core and PANI shell onto a planar Au mirror (Figure 7a). This setup results in a strong coupling of light fields in the gap between the Au core and the Au mirror, referred to as the “hot spot” (Figure 7b). This hot spot leads to a strong additional coupled resonance, which can be tuned by changes in the surrounding optical environment, thereby altering the plasmon resonance. Thus, when the voltage is swept from −0.2 V to 0.6 V at a scanning speed of 50 mV/s, the redox state of the PANI shell changes, causing its effective refractive index to vary by 0.6. This results in the spectral scattering peak shifting from 642 nm to 578 nm and a corresponding color change from red to green (Figure 7c).

The integration of plasmonic materials with EC molecules and polymers can also improve the stability of EC materials. Wu et al. [104] prepared a dual-band EC film composed of a two-dimensional ordered macroporous (2DOM) Au LSPR layer and a poly(3,4-(2,2-dimethylpropyl dioxy) thiophene) (PProDot-Me_2_) EC layer. The large holes in the 2DOM Au structure enable the LSPR effect to occur not only in the visible region but also in the near-infrared region (Figure 7d and e). Importantly, the 2DOM-Au/PProDot-Me_2_ films exhibit superior cyclic stability, with transmittance modulation at 580 nm and 1800 nm decreasing by only 14.2 % and 15 %, respectively, after 10,000 cycles. In contrast, the transmittance modulation of PProDot-Me_2_ films at 580 nm and 1800 nm decreased by 37.7 % and 17 %, respectively (Figure 7f). This enhanced cyclic stability results from incident light exciting electron pairs and hot hole pairs on the surface of 2DOM Au, with positively charged hot holes further interacting with PProDot-Me_2_. This interaction makes negatively charged ClO_4_^−^ ions more easily attracted by electromagnetic forces to the PProDot-Me_2_ layer. Additionally, the 2DOM-Au/PProDot-Me_2_ films exhibit better ion diffusion performance, minimizing the concentration of chlorine ions, which had previously been found to degrade transmittance modulation [105]. Compared to the 2.49 % chlorine concentration in the PProDot-Me_2_ film, the chlorine concentration of the 2DOM-Au/PProDot-Me_2_ film was only 1.77 % after 10,000 cycles. Hence, integration with plasmonic materials enhances the cyclic stability of EC molecules and polymers.

**Figure 8:**
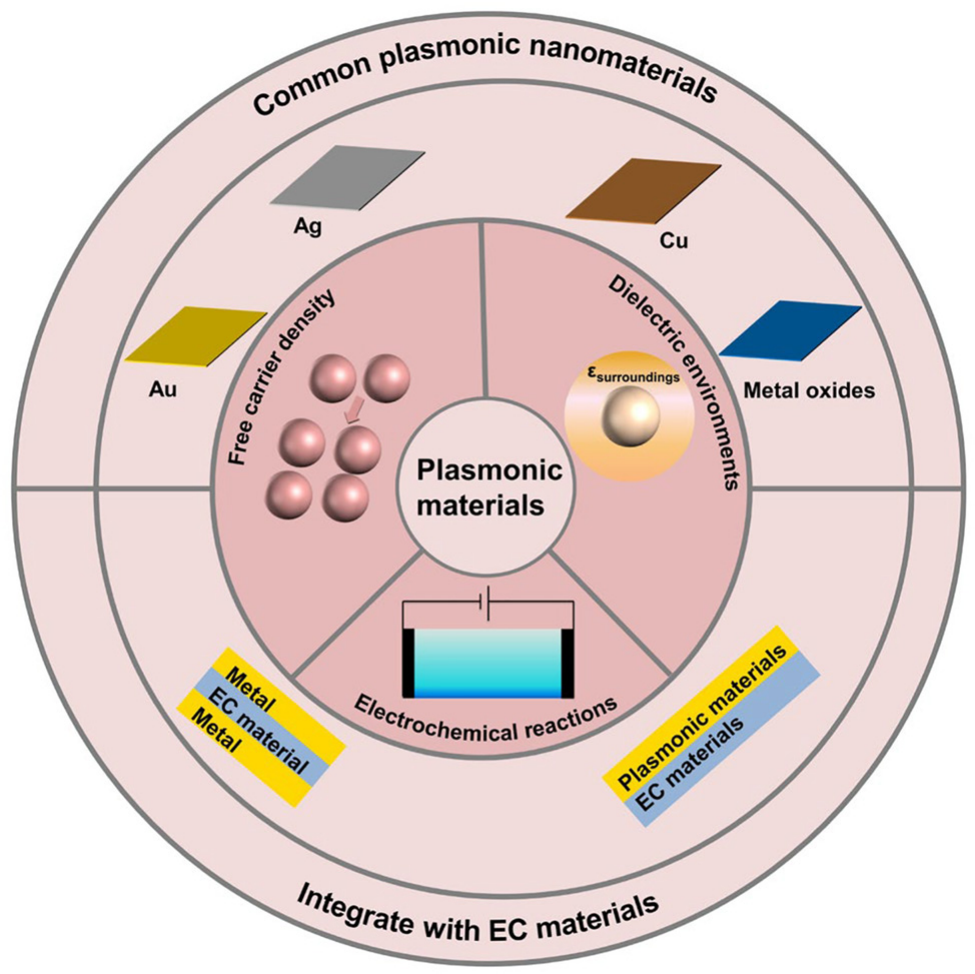
Summary schematic diagram of typical plasmonic EC materials and their applications in electrochromic technology.

## 5 Conclusion and perspective

In conclusion, LSPR in plasmonic nanomaterials represents a captivating optical phenomenon with substantial potential across various fields. Ongoing advancements in plasmonic materials and technologies have paved the way for their application in metasurface films, optical materials, dynamic light modulation, and photocatalysis. Plasmonic electrochromism has emerged as a key area of interest for the development of optoelectronic devices. According to the Drude–Lorentz model, plasmonic EC processes are primarily associated with non-Faradaic reactions. Resonances induced by ion adsorption on the material’s surface lead to distinctive light absorption, enabling a broader color palette and improved cyclic stability in electrochromism.

This review has provided an overview of various mechanisms of plasmonic electrochromism, including the Drude–Lorentz model, Faradaic reactions, non-Faradaic reactions, and the influence of the dielectric environment (Figure 8). In plasmonic electrochromism, these mechanisms are intertwined, and a comprehensive understanding often requires considering the combined effects of multiple mechanisms. The development and applications of commonly used plasmonic EC materials were also discussed, highlighting their potential in achieving stable and high-performance EC devices. However, several challenges remain to be addressed in order to further enhance the performance of plasmonic EC devices and expedite their practical applications.

1. Mechanism investigation. Deeper research into the mechanisms of plasmonic electrochromism is needed, including more extensive quantitative studies. Quantitative descriptions of how electrochemical reactions modulate carrier concentration are essential. A comprehensive understanding of the electrochemical and optical properties of hybrid materials is necessary to enhance plasmonic EC performance.
2. Material optimization. Optimizing the structure and composition of plasmonic materials is crucial to improve the stability of plasmonic EC devices and achieve full-color gamut devices. Finding cost-effective alternatives to noble metals like Au and Ag, such as Cu, is essential for enabling widespread applications in industrial and commercial settings. Irregularities in size and shape, coupled with the lack of effective control, contribute to the broadening of the surface plasmon resonance, impacting the overall performance of the EC applications involving Cu [106]. To address this issue, we acknowledge the necessity for a more in-depth exploration into methods for precisely regulating the size and shape of Cu nanocrystals. Achieving a higher degree of uniformity in these aspects is pivotal to enhancing the performance and stability of Cu-based EC devices. Additionally, the challenge of insufficient control over interparticle distances is an important consideration, and a thorough investigation into strategies to achieve better control in this regard is crucial for optimizing the EC behavior of Cu materials. This aspect will be duly emphasized in future work and will contribute to the advancement of the field.
3. Integration with advanced technologies. Plasmonic devices need to be integrated with other advanced technologies to expand their functionalities and application scenarios. For example, integrating plasmonic EC windows can convert incoming visible light into heat energy for building heating while providing light control. The great application potential of plasmonic EC materials in domains such as camouflage, display, thermal management, photothermal therapy, and biosensors is thoroughly investigated [7], [8]. Their versatility in addressing real-world challenges is highlighted, and providing a comprehensive overview of the practical implications. Furthermore, their role in systems involving plasma–exciton–exciton interactions is explored, introducing controlled modulation of light emission and opening up new possibilities [107]. Additionally, the potential applications of near-infrared tunable surface plasma absorption in waveguide modulators and long distance communication wavelength switches are explored [33]. This insight is crucial in understanding the materials’ practical implications for the telecommunications sector, adding another layer to the commercial considerations. Furthermore, plasmonic devices can be integrated with EC technology to create plasmonic EC displays, combining plasmonic multicolor capabilities with the energy-efficient bistable properties of ECs [108].

With continued research and improved performance, plasmonic EC devices are expected to find widespread applications in various aspects of people’s lives, offering innovative solutions in areas such as smart windows, displays, and energy-efficient technologies.
